# Antimicrobial-Resistant *Escherichia coli* Strains and Their Plasmids in People, Poultry, and Chicken Meat in Laos

**DOI:** 10.3389/fmicb.2021.708182

**Published:** 2021-07-26

**Authors:** Aline I. Moser, Esther Kuenzli, Edgar I. Campos-Madueno, Thomas Büdel, Sayaphet Rattanavong, Manivanh Vongsouvath, Christoph Hatz, Andrea Endimiani

**Affiliations:** ^1^Institute for Infectious Diseases, University of Bern, Bern, Switzerland; ^2^Department of Public Health, Epidemiology, Biostatistics and Prevention Institute, University of Zurich, Zurich, Switzerland; ^3^Swiss Tropical and Public Health Institute, Basel, Switzerland; ^4^University of Basel, Basel, Switzerland; ^5^Microbiology Laboratory, Mahosot Hospital, Vientiane, Laos; ^6^Division of Infectious Diseases and Hospital Epidemiology, Kantonsspital St. Gallen, St. Gallen, Switzerland

**Keywords:** *mcr-1*, *mcr-3*, CTX-M, *E. coli*, Laos, gut colonization, poultry, meat

## Abstract

Antimicrobial resistant (AMR) Enterobacterales are widely distributed among the healthy population of the Indochinese peninsula, including Laos. However, the local reservoir of these pathogens are currently not known and possible sources such as agricultural settings and food have rarely been analyzed. In this work, we investigated the extended-spectrum cephalosporin- (ESC-) and colistin-resistant *Escherichia coli* strains (CST-R-*Ec*) isolated from the gut of local people, feces of poultry, and from chicken meat (60 samples each group) in Laos. Whole-genome sequencing (WGS) analysis based on both short- and long-read sequencing approaches were implemented. The following prevalence of ESC-R-*Ec* and CST-R-*Ec* were recorded, respectively: local people (70 and 15%), poultry (20 and 23.3%), and chicken meat (21.7 and 13.3%). Core-genome analysis, coupled with sequence type (ST)/core-genome ST (cgST) definitions, indicated that no common AMR-*Ec* clones were spreading among the different settings. ESC-R-*Ec* mostly possessed *bla*_CTX–M–15_ and *bla*_CTX–M–55_ associated to IS*Ecp1* or IS*26*. The majority of CST-R-*Ec* carried *mcr-1* on IncX4, IncI2, IncP1, and IncHI1 plasmids similar or identical to those described worldwide; strains with chromosomal *mcr-1* or possessing plasmid-mediated *mcr-3* were also found. These results indicate a high prevalence of AMR-*Ec* in the local population, poultry, and chicken meat. While we did not observe the same clones among the three settings, most of the *bla*_CTX–Ms_ and *mcr-1/-3* were associated with mobile-genetic elements, indicating that horizontal gene transfer may play an important role in the dissemination of AMR-*Ec* in Laos. More studies should be planned to better understand the extent and dynamics of this phenomenon.

## Introduction

Gut colonization with extended-spectrum cephalosporin-resistant Enterobacterales (ESC-R-Ent) is a major health threat worldwide, as these bacteria represent a risk for subsequent difficult-to-treat extraintestinal infections (Karanika et al., 2016). Moreover, the recent emergence of colistin-resistant Enterobacterales (CST-R-Ent) represents an additional concern (Nordmann and Poirel, 2016).

Previous studies have indicated a high prevalence of ESC-R-Ent in the healthy population of Laos (Nakayama et al., 2015; Stoesser et al., 2015). In contrast, prevalence data of CST-R-Ent in the Laotian population is lacking, although sporadic isolates have been reported (Olaitan et al., 2015; Malchione et al., 2019). The environmental reservoir of these antimicrobial resistant (AMR) bacteria in Laos is unclear since possible sources such as agricultural settings and food have rarely been examined (Olaitan et al., 2015). While studies from surrounding countries indicated high prevalence rates of ESC-R *Escherichia coli* (ESC-R-*Ec*) in poultry and chicken meat, data for Laos does not exist (Ueda et al., 2015; Nguyen Do et al., 2016; Tansawai et al., 2019b). Moreover, little is known about the possible transfer of AMR pathogens from these reservoirs to humans (Ueda et al., 2015). In this context, a multifaceted One Health approach using state-of-the-art molecular methodologies is essential to understand and try limiting the spread of antimicrobial resistance (Kim and Cha, 2021). However, studies implementing whole-genome sequencing (WGS) to characterize AMR-*Ec* collected simultaneously from different settings and in the same region are still lacking.

In a recent study performed by our group in Tanzania, we identified chicken meat, poultry, fish and water as responsible for the transmission of specific AMR-*Ec* to humans. In that study, our conclusions were supported by a solid molecular comparison of strains obtained by implementing a core-genome analysis based on the Illumina short-read sequencing output (Moser et al., 2020).

In the present work, we applied a similar approach to compare ESC-R- and/or CST-R-*Ec* found in local people, poultry, and chicken meat. In addition, strains were compared to those from a small sample of Swiss travelers that visited the country. We also performed a further combined short- and long-read WGS approach to characterize and compare the mobile genetic elements (MGEs) found in such AMR-*Ec* strains.

## Materials and Methods

### Volunteers and Samples Collection

We studied samples from local people, poultry, and retailed chicken meat from Laos together with those from Swiss residents who traveled to Laos. In particular, 60 volunteers (≥ 18-year-old) working in 12 hotels located in Vientiane (Laos) were recruited during August-October 2018. Volunteers who signed a written informed consent form and filled in an epidemiological questionnaire provided self-collected rectal swabs in transport medium (Copan).

In addition, a small set of samples from Swiss volunteers (≥18-year-old; *n* = 9), who were planning to travel to Laos and received pre-trip medical health advice at the travel clinics based in Basel (Swiss Tropical and Public Health Institute), Zurich (Epidemiology, Biostatistics and Prevention Institute of the University of Zurich), and Aarau (Department of Infectious Diseases and Hospital Hygiene, Aarau Cantonal Hospital) between August 2018 and May 2019, were included in the analysis. Volunteers provided self-collected rectal swabs (Copan) and filled out epidemiological questionnaires in the week before and the week after traveling. Samples were received and processed by the Institute for Infectious Diseases (IFIK; Bern, Switzerland) within 24 h after collection.

Fecal samples from poultry (*n* = 60) were collected during August 2018 from 7 different farms using transport swabs (Copan). Raw chicken meat (*n* = 60) was obtained from 24 markets during August 2018. For each chicken meat sample, 5 g was cut off and stored in transport swabs (Copan). Both farms and markets were located in and around Vientiane.

All swabs collected in Laos were processed at the Microbiology Laboratory, Mahosot Hospital (MLMH) in Vientiane within 24 h after collection and strains growing on selective agar plates (see below) were shipped via courier to the IFIK for further analyses.

Ethics approval was obtained from the Kantonale Ethikkommission Zürich, the Ethics Committee Nordwest- und Zentral-Schweiz, and the Lao National Ethics Committee for Health Research (BASEC #: 2017-01945, NECHR #:2018-033). A written informed consent was obtained from all participants.

### Samples Processing

At both MLMH and IFIK, samples were enriched overnight in LB broth containing cefuroxime (3 mg/L) or CST (2 mg/L). Carbapenem-, ESC-, and/or CST-R-Ent were screened by plating the enrichments on selective ChromID ESBL/Carba or ChromID Colistin R plates (bioMérieux) (Moser et al., 2020). All colonies showing different colors were selected for species identification (ID).

### Phenotypic Characterization

Identification was achieved at the IFIK using the MALDI-TOF MS (Bruker). MICs for antibiotics were obtained by using the Sensititre GNX2F microdilution panel (Thermo Fisher Scientific) and results were interpreted according to the EUCAST breakpoints (v.9.0, 2019).^1^

Since in the overall study only 2 CST-R *Klebsiella pneumoniae* were found (data not shown), only *Ec* strains were considered for further analyses. For simplicity, in the present work, the acronym “AMR-*Ec*” was used to define ESC- R-, CST-R-*Ec*, and/or carbapenem-R-*Ec* strains.

### Detection of the Main Antimicrobial Resistance Genes and Analysis of Clonality

AMR-*Ec* were analyzed for the main *bla* and *mcr-1/-2* genes using the CT103XL microarray (Check points) (Bernasconi et al., 2017; Budel et al., 2020). Results for *bla*_CTX–Ms_ were categorized as “group” or “subgroup,”^2^ while those for *mcr* genes as “-like.” PCR-based approaches were implemented to detect *mcr-1* to *mcr-8* in all strains showing a colistin MIC > 0.25 mg/L (Budel et al., 2020).

To analyze the clonality of isolates, the repetitive extragenic palindromic PCR (rep-PCR) characterization was performed as previously described (Budel et al., 2019, 2020). BioNumerics 4.5 was used to construct homology trees and clones were defined as such if they showed ≥85% similarity.

### Whole-Genome Sequencing

DNA extracted using the PureLink^TM^ Microbiome DNA Purification kit (Thermo Fisher Scientific) was used for WGS performed with the NovaSeq 6000 (Illumina) sequencer (2 × 150 bp reads). Assemblies (SPAdes, v3.12.0) were deposited on NCBI under BioProjects PRJNA667896, PRJNA667852, and PRJNA667861 (isolates from local sources, travelers pre-trip, and travelers post-trip, respectively).

All assemblies were analyzed with the tools of the Center for Genomic Epidemiology (CGE)^3^ to define sequence type (ST), antimicrobial resistance genes (ARGs), and plasmid replicons. The Illumina raw reads were used to determine the core-genome ST (cgST) using the CGE. Assemblies also underwent core-genome single nucleotide variant (SNV) analysis as previously described using Parsnp v.1.2 (Budel et al., 2020; Moser et al., 2020). Strain EH6-18-27-B was randomly selected as reference genome and the SNV tree was visualized with iTOL.^4^

Selected isolates were additionally sequenced on a MinION using the SQK-RBK004 sequencing kit and FLO-MIN106D (R9) flow cells (Oxford Nanopore). Raw reads were trimmed using Porechop (v.0.2.4) to remove sequencing adaptors and assembled using Flye (v.2.7.1). The assemblies were polished using Pilon (v.1.22) with the trimmed Illumina reads (Trimmomatic v.0.36) (Campos-Madueno et al., 2020). Annotation was obtained with the NCBI Prokaryotic Genome Annotation Pipeline.

Hybrid assemblies were deposited on NCBI under BioProject PRJNA670073 and PRJNA670071 (isolates from local sources and travelers, respectively). Plasmid sequences were compared using BLASTn and visualized with the BLAST Ring Image Generator (BRIG) (v.0.95).

### Limiting Costs

To limit costs, only one *Ec* colony underwent phenotypic characterization (with the exception of travelers’ samples where 5 colonies were tested). Moreover, due to the still high number of AMR-*Ec* detected in the overall study (*n* = 109), only subsets of representative strains were analyzed with the above-mentioned molecular methodologies: 67 strains (61.5%) were analyzed with rep-PCR, microarray, and PCRs, while 49 (45%) and 17 (15.6%) underwent WGS by using Illumina alone and both Illumina and Nanopore, respectively (Supplementary Table 1). In particular, the 67 out of 109 AMR-*Ec* were selected based on their phenotypic profiles, while the 49 intended for Illumina were selected based on their rep-PCR clone and their *bla*/*mcr* genes.

## Results and Discussion

The main scope of this work was to evaluate the spread and extent of AMR-*Ec* in several key settings in Laos, but also to explore the possible transmission of these pathogens to the intestinal tract of foreign people visiting the country. To do this, a large collection of AMR-*Ec* strains recovered from local sources and international travelers was studied.

### Colonization Prevalence of AMR-*Ec* in Local People

Sixty local people (overall mean age: 31.5 years) in the community were enrolled in the study. Of note, 36 (60%) of them had received antibiotic treatment and 8 (13.3%) had been hospitalized in the last 12 months (Table 1).

**TABLE 1:** Demographic and clinical characteristics of the 60 local (Laos) people in the community enrolled in the present study.

Employee	Sex/age	Hotel #	Use of antibiotics in the past 12 months^a^	Traveled abroad in the past 12 months (days)^b^	Hospitalized in Laos^c^	*E. coli* strains detected in the present work (phenotype)
01	F/32	1	Yes, AMP (4/3)	No	No	None
02	F/54	1	No	No	No	EH01-18-02 (CST-R)
03	F/27	1	Yes, na (16/14)	No	No	EH01-18-03 (CST-R)
04	M/34	1	Yes, AMX (na/3)	No	No	EH01-18-04-A (ESC-R)
05	F/42	1	Yes, AMX (na/3)	No	No	EH01-18-05 (ESC-R)
06	M/36	2	Yes, na (16/3)	No	Yes (4/3)	EH01-18-06 (ESC-R)
07	F/46	2	Yes, AMX (na/3)	No	No	EH02-18-07 (ESC-R)
08	F/na	2	No	No	No	EH02-18-08 (ESC-R)
09	F/33	2	Yes, AMX (2/2)	No	No	None
10	F/28	2	No	No	No	EH02-18-10 (ESC-R)
11	F/34	3	Yes, AMP (12/3)	No	No	EH03-18-11 (ESC-R)
12	F/19	3	No	No	No	EH03-18-12 (ESC-R)
13	M/25	3	Yes, AMP (8/3)	No	No	EH03-18-13 (ESC-R)
14	F/20	3	Yes, AMX (4/1)	No	No	EH03-18-14 (ESC-R)
15	F/23	3	Yes, na (na/2)	No	Yes (3/2)	None
16	M/39	4	No	No	No	EH04-18-16 (ESC-R)
17	F/21	4	No	No	No	EH04-18-17 (ESC-R)
18	F/30	4	Yes, AMX (8/2)	No	No	EH04-18-18 (CST-R)
19	M/28	4	No	No	No	EH04-18-19 (ESC-R)
20	F/23	4	No	No	No	None
21	F/30	5	No	Singapore (na), Indonesia (na), Malaysia (na), and Thailand (na)	Yes (6/1)	None
22	M/22	5	Yes, AMX (28/1)	No	No	EH5-18-22 (ESC-R)
23	F/21	5	Yes, na (12/1)	No	Yes (3/1)	EH5-18-23 (ESC-R)
24	F/32	5	No	No	No	EH5-18-24 (ESC-R)
25	F/18	5	No	No	No	EH5-18-25 (ESC-R)
26	F/35	6	Yes, na (1/7)	No	No	EH6-18-26 (ESC-R)
27	F/34	6	Yes, AMX (na/na)	No	No	EH06-18-27-B (CST-R)
28	F/49	6	Yes, AMX (1/3)	No	No	EH06-18-28 (ESC-R)
29	F/19	6	Yes, AMX (20/7)	No	Yes (11/4)	EH06-18-29 (ESC-R)
30	F/24	6	Yes, AMX (na/7)	Thailand (3)	No	EH06-18-30 (ESC-R)
31	M/36	7	Yes, na (24/2)	No	No	EH7-18-31 (ESC-R)
32	F/24	7	Yes, AMX (12/3)	No	No	None
33	F/18	7	Yes, na (na/2)	No	No	None
34	F/25	7	Yes, AMX (na/3)	Thailand (3)	No	EH7-18-34 (ESC-R)
35	F/30	7	Yes, AMP (44/5)	No	No	EH07-18-35 (ESC-R)
36	M/20	7	No	No	No	EH08-18-36 (ESC-R)
37	M/24	7	No	No	Yes (1/3)	EH08-18-37 (ESC-R)
38	F/36	8	No	No	No	EH08-18-38 (ESC-R)
39	M/32	8	Yes, na (32/3)	No	No	EH08-18-39 (ESC-R)
40	F/35	8	No	No	No	EH08-18-40 (ESC-R)
41	M/52	9	No	No	No	EH09-18-41 (CST-R)
42	F/40	9	Yes, AMX (8/3)	No	No	EH09-18-42 (ESC-R)
43	F/39	9	Yes, na (na/7)	Thailand (3)	No	None
44	M/41	9	No	No	No	EH09-18-44 (ESC-R)
45	M/25	9	No	Vietnam (8) and Thailand (1)	No	EH09-18-45 (ESC-R)
46	F/43	10	No	No	No	EH10-18-46 (CST-R)
47	F/47	10	Yes, AMX (na/4)	No	No	EH10-18-47 (CST-R)
48	F/32	10	Yes, AMX (na/3)	No	No	EH10-18-48 (ESC-R)
49	F/23	10	Yes, AMX (12/3)	No	No	EH10-18-49 (ESC-R)
50	F/27	10	Yes, na (na/7)	No	Yes (na/7)	EH10-18-50 (ESC-R)
51	M/41	11	Yes, AMX (4/7)	No	No	None
52	M/30	11	No	No	No	EH11-18-52 (ESC-R)
53	F/20	11	No	No	No	EH11-18-53 (ESC-R)
54	F/48	11	Yes, AMX (24/5)	No	No	EH11-18-54 (ESC-R)
55	F/32	11	No	No	No	EH11-18-55 (CST-R)
56	F/32	12	Yes, na (10/1)	No	No	EH12-18-56 (CST-R)
57	F/39	12	No	Thailand (90)	No	EH12-18-57 (ESC-R)
58	F/34	12	No	No	Yes (1/3)	EH12-18-58 (ESC-R)
59	F/na	12	Yes, na (na/7)	No	No	EH12-18-59 (ESC-R)
60	F/23	12	Yes, na (na/1)	No	No	EH12-18-60 (ESC-R)

*F, female; M, male; na, not available; AMP, ampicillin; AMX, amoxicillin; CST-R, colistin-resistant; ESC-R, extended-spectrum cephalosporin-resistant.*

*^*a*^If yes, antibiotic (weeks before sampling/total days of treatment).*

*^*b*^It includes only trips for >2 days.*

*^*c*^If hospitalized (months before sampling/days hospitalized).*

Fifty-one (85%) subjects resulted colonized at gut level with AMR-*Ec*; in particular, 42 (70%) carried ESC-R-*Ec*, while 9 (15%) possessed CST-R-*Ec*. The high prevalence observed for ESC-R-*Ec* was consistent with previously reported data (72%) in the Laotian healthy population in 2012 (Nakayama et al., 2015). On the other hand, though sporadic isolates were already reported in Laos (Malchione et al., 2019), prevalence data about the carriers of CST-R-*Ec* was not available. A recent study in Vietnam (2017–2018) found a colonization prevalence with CST-R-*Ec* of 70% among 98 people living in a rural community (Yamamoto et al., 2019), but we are unable to explain this significant difference with our data.

### AMR-*Ec* Strains in Poultry and Chicken Meat

In the present study, the prevalence of AMR-*Ec* colonizing poultry and contaminating chicken meat in Laos was analyzed for the first time. Therefore, figures from the countries surrounding Laos were used for comparison.

Twenty-six (43.3%) out of 60 poultry fecal samples resulted positive for AMR-*Ec*, of which 12 (20%) harbored ESC-R-*Ec* and 14 (23.3%) had CST-R-*Ec*. The prevalence of ESC-R-*Ec* was analogous to that reported for Thailand (26%), but lower than the one observed in Vietnam (43%) during 2013–2014 (Ueda et al., 2015; Tansawai et al., 2019a). With regard to the CST-R-*Ec*, a study from Vietnam reported a lower prevalence (9%), but strains were detected in 2011–2012 (Vounba et al., 2019).

Of the 60 chicken meat samples analyzed, 17 (28.3%) tested positive for AMR-*Ec*. In particular, 13 (21.7%) samples harbored ESC-R-*Ec* [of which 5 (38.5%) also CST-R], 8 (13.3%) possessed CST-R-*Ec* (of which 5 also ESC-R), and one (2.0%) had a carbapenem-R-*Ec*. The prevalence of ESC-R-*Ec* observed in our study was considerably lower than that reported in Thailand (70%) and Vietnam (93%) (Nguyen Do et al., 2016; Tansawai et al., 2017). Unfortunately, data about the prevalence of CST-R-*Ec* from surrounding countries are still lacking. However, as we observed, a recent study from Vietnam reported that 39% of the ESC-R-*Ec* from chicken meat were also CST-R (Yamaguchi et al., 2018). The observation that ∼40% of the ESC-R-*Ec* contaminating chicken meat in Laos and Vietnam is also CST-R is alarming and should drive the implementation, whenever possible, of Hazard Analysis and Critical Control Points concepts to avoid the spread of these pathogens through the food chain (Silva et al., 2018).

### Gut Colonization With AMR-*Ec* in Travelers

In addition to the samples obtained in Laos, 9 travelers (overall mean age: 50.8 years) from Switzerland who visited Laos for at least 8 days were enrolled in our study. Notably, all of them traveled to other countries during the last year, but none had visited Laos before (Supplementary Table 2).

Five out of the 9 travelers (55.6%) returned home colonized with AMR-*Ec*. In total, 10 isolates (5 ESC- R-, 4 CST-R, and one ESC-/CST-R-*Ec*) were found (Table 2). Of note, two travelers (i.e., 49 and BS115; 22.2%) were already colonized with CST-R-*Ec* before the trip to Laos. Despite the low number of participating volunteers in the present study, the pre- and post-trip prevalence of AMR-*Ec* colonization recorded were consistent with previous surveys on Swiss and European travelers who visited South Asia (Kuenzli et al., 2014; Bernasconi et al., 2016; Arcilla et al., 2017; Frost et al., 2019; Pires et al., 2019; Schaumburg et al., 2019; Kantele et al., 2021).

**TABLE 2:** Phenotypic and molecular features of the 49 *E. coli* that underwent WGS isolated from rectal swabs of local residents (*n* = 11), chicken stool (*n* = 12), chicken meat (*n* = 14), and 9 travelers visiting Laos (pre-trip *n* = 2; post-trip *n* = 10).

Strain^a^	Resistant MIC (mg/L)^b^	ST	cgST	Antimicrobial resistance genes (ARGs)^c,d^	Replicon type(s)^c,e^
**Local people**					
EH01-18-02	SXT (>4), DOX (16), CST (>4)	4014	90921	*mcr-1.1*, *bla*_TEM–1B_, *aadA1, aadA2, mdf(A), cmlA1, qnrS1, sul3, tet(A), dfrA12*	FIA, R
EH01-18-04-A	CTX (> 32), FEP (4), CIP (0.5), SXT (> 4), DOX (8), TGC (0.5)	131	135528	*bla*_CTX–M–55_, *bla*_TEM–1B_, *aadA5, aph(3″)-lb, aph(6)-ld, mph(A), sul1, sul2*, *tet(A), dfrA17*	FIA, FIB, FII
EH02-18-07	CTX (> 32)	3489	86381	*bla*_CTX–M–15_, *mdf(A), qnrS1, tet(A)*	FII
EH04-18-18	CTX (16), CIP (0.5), DOX (16)	5215	95817	*mcr-1.1, bla*_OXA–10_, *aac(3)-IId, aadA2, aph(3′)-Ia, erm(B), mdf(A), mph(A), qnrS1, arr-2, sul3, dfrA12, dfrA14*	FIA(HI1), FIB(K), FII, Y
EH06-18-27	SXT (>4), DOX (8), CST (4)	9555	97044	*mcr-1.1, bla*_TEM–1B_, *aadA1, mdf(A), cmlA1, qnrS13, sul3, dfrA15*	FIA(HI1), FIB(K), p0111
EH08-18-36	CTX (32), GEN (>8), SXT (>4), DOX (8)	542	122545	***bla*_CTX–M–55_**, *aac(3)-IId, aadA24, mdf(A), mef(B), floR, qnrS1, sul2, sul3, tet(A), tet(M), dfrA12*	FIA, **HI1** (273 kb),
EH09-18-41	CIP (>2), GEN (>8), SXT (>4), DOX (8), CST (4)	3944	32146	***mcr-3.1***, *bla*_TEM–1B_, *aac(3)-IId, aadA1, aadA2, mdf(A), cmlA1, floR, sul2, sul3, tet(A), tet(M), dfrA12*	**FIB** (132 kb), Q1, X1, Y
EH10-18-47	SXT (>4), DOX (8), CST (4)	165	40030	*mcr-1.1*, *bla*_TEM–1B_, *aph(3″)-Ib, aph(6)-Id, mdf(A), floR, qnrS1, sul2, tet(A), dfrA14*	p0111
EH10-18-50	CTX (>32), FEP (4), SXT (>4), DOX (8)	226	81270	*bla*_CTX–M–15_, *bla*_TEM–1A_, *aadA5, aph(3″)-Ib, aph(3′)-Ia, aph(6)-Id, mdf(A), floR, qnrS1, sul2, tet(A), dfrA17*	FIB, FII
EH11-18-55	SXT (<4), CST (>4)	175	31835	***mcr-1.1***, *aadA1, mdf(A), qnrS1, sul3, dfrA14*	FIA, FIB, **HI1** (227 kb), p0111
EH12-18-60	CTX (8), TGC (0.5)	1081	90277	*bla*_CTX–M–14_, *aph(3″)-lb, mdf(A)*	X1, Y
**Poultry**					
EF1-18-01	CTX (>32), FEP (16), CIP (>2), GEN (>8), SXT (>4), DOX (8)	1011	111265	*bla*_CTX–M–55_, *bla*_TEM–1B_, *aadA2, aph(3″)-lb, aph(6)-ld, mph(A), sul1, sul2, tet(A), dfrA12*	nd
EF1-18-06-B	CTX (>32), FEP (8), CIP (>2), GEN (>8), SXT (> 4), DOX (8)	162	131640	*bla*_CTX–M–9–like_, *aph(3″)-lb, aph(6)-ld, mdf(A), sul2, tet(A)*	Col156, l2(Delta), p0111
EF2-18-09	DOX (8), CST (>4)	1630	39790	***mcr-1.1***, *bla*_TEM–1B_, *aadA1, mdf(A), qnrS1, tet(A), dfrA1*	**X4** (33 kb), p0111
EF3-18-22	GEN (>8), DOX (16), CST (>4)	11090	137946	*mcr-1.1*, *bla*_TEM–1B_, *aac(3)-IId, mdf(A), qnrS13, tet(A)*	FIB, p0111, Col156
EF4-18-32	CIP (>2), GEN (>8), SXT (>4), CST (4)	2179	32164	*mcr-1.1, aac(3)-IId, mdf(A), sul3, dfrA14*	FIB, FIC, p0111
EF5-18-41	CIP (>2), GEN (>8), SXT (>4), DOX (>16), CST (>4)	69	32650	***mcr-1.1***, *bla*_TEM–1B_, *aac(3)-IId, aadA1, aadA2, mdf(A), cmlA1, floR, sul2, sul3, tet(A), tet(M), dfrA12*	FIA, FIB, I2, **P1** (57 kb), B/O/K/Z, Col156
EF7-18-51	CIP (>2), GEN (>8), SXT (>4), DOX (16), CST (>4)	48	39128	***mcr-1.1***, *bla*_TEM–1B_, *aac(3)-IId, aadA1, aadA2b, aph(3″)-Ib, aph(6)-Id, mdf(A), cmlA1, sul3, tet(A), dfrA15*	**X4** (33 kb), ColE10, p0111
EF7-18-53	CTX (32), CIP (>2), DOX (>16)	4981	38972	*bla*_CTX–M–15_, *aph(3″)-Ib, aph(6)-Id, mdf(A), tet(A), dfrA15*	FIB, p0111
EF7-18-54	CTX (32), CIP (>2), DOX (16)	4981	38972	*bla*_CTX–M–15_, *aph(3″)-Ib, aph(6)-Id, mdf(A), tet(A, dfrA15*	FIB, p0111
EF7-18-55	CTX (8), CIP (>2), GEN (>8), SXT (>4), DOX (16)	155	129299	*bla*_CTX–M–55_, *aac(3)-IId, aadA1, aadA2b, mdf(A), catA2, cmlA1, qnrS1, sul3, tet(A), dfrA15*	FII, FIB, p0111
EF7-18-58	CTX (8), CIP (>2), GEN (>8), SXT (>4), DOX (16)	155	129299	***bla*_CTX–M–55_**, *aac(3)-IId, aadA1, aadA2b, mdf(A), catA2, cmlA1, qnrS1, sul3, dfrA15*	FIB(K), **FII** (82 kb), p0111
EF7-18-60	GEN (>8), SXT (>4), DOX (8), CST (4)	7352	44820	*mcr-1.1, bla*_TEM–1–like_, *aadA1, aph(6)-ld, mdf(A), cmlA1, sul3, dfrA12*	FIB(K), FII(pCoo), p0111
**Chicken meat**					
EM03-18-06	CTX (32), FEP (4), CIP (>2), GEN (>8), SXT (>4), DOX (8), CST (4)	617	8237	*bla*_CTX–M–55_, ***mcr-1.1***, *aph(3″)-lb, aph(6)-ld, aac(3)-IId, aadA2, mdf(A), qnrS1, sul2, sul3, tet(A), tet(B), dfrA12*	FIB(A P001918), FII, X1, **X4** (33 kb)
EM03-18-08	CIP (>2), GEN (4), SXT (>4), DOX (8), CST (4)	10	39835	***mcr-3.5*,** *bla*_TEM–1B_, *aac(3)-IV, aadA1, aadA2, aph(3′)-Ia, aph(4)-Ia, mdf(A), mef(B), cmlA1, floR, sul2, sul3, tet(A), dfrA12*	FIA, FIB, **FII** (77 kb)
EM06-18-14	TZP (>64), CTX (>32), FEP (>16), ETP (>4), CIP (>2), GEN (>8), SXT (>4), DOX (>16)	156	87293	***bla*_*NDM–*5_**, *bla*_CTX–M–65_, *bla*_TEM–1B_, *aac(3)-IV, aadA2, aph(3′)-Ia, aph(4)-Ia, rmtB, fosA3, mdf(A), floR, sul1, sul2, tet(B), dfrA12*	**FII** (91 kb)
EM07-18-20	CTX (16), CIP (>2), SXT (>4), DOX (16), TGC (1)	162	39191	*bla*_CTX–M–65_, *bla*_TEM–1B_, *aadA5, aph(3″)-Ib, aph(6)-Id, fosA3, mdf(A), mph(A), floR, sul1, sul2, dfrA17*	FIB, FIC, I2
EM07-18-22	CTX (>32), CIP (2), SXT (>4)	2179	122779	*bla*_CTX–M–65_, *bla*_OXA–1_, *aac(6′)-lb-cr*, *mdf(A), catB3, arr-3, dfrA17*	FIB(AP001918), FIC(FII), Y
EM08-18-25	CTX (8), GEN (8), SXT (>4), DOX (16)	23	56751	*bla*_CTX–M–65_, *bla*_TEM–1B_, *aac(3)-IV, aadA1, aph(3″)-Ib, aph(4)-Ia, aph(6)-Id, fosA3, mdf(A), floR, sul1, sul2, dfrA1*	FIB, FIC, I1-I(Gamma), Q1
EM09-18-27	CTX (32), FEP (4), SXT (>4), DOX (8)	226	81270	*bla*_CTX–M–15_, *bla*_TEM–1A_, *aadA5, aph(3″)-Ib, aph(6)-Id, mdf(A), floR, qnrS1, sul2, tet(A), dfrA17*	FIB, FII
EM10-18-28	SXT (>4), DOX (>16), TGC (0.5), CST (>4)	648	39741	***mcr-1.1***, *bla*_TEM–1A_, *mdf(A), floR, qnrS1, sul2, tet(A), tet(B), dfrA14*	**P1** (48 kb), p0111
EM10-18-30	CTX (>32), FEP (16), CIP (>2), SXT (>4), DOX (16), CST (>4)	2505	39108	*bla*_CTX–M–55_, *bla*_TEM–1B_, ***mcr-1.1***, *aadA1, aph(3″)-lb, aph(&)-ld, mdf(A), sul1, sul2, dfrA1*	FIB(A P001918), FIC(FII), 1-I(Gamma), **l2(Delta)** (63 kb)
EM18-18-42	CTX (2), DOX (16), TGC (0.5), CST (>4)	11225	47189	***bla*_CTX–M–14_**, ***mcr-1.1***, *mph(A), qnrS1, tet(A)*	**HI1** (210 kb), X1
EM20-18-50	SXT (>4), CST (>4)	1585	31409	***mcr-1.1***, *mdf(A), qnrS1, sul2, tet(A), dfrA14*	FIB(K), **P1** (48 kb), p0111
EM22-18-53	CTX (8), CIP (>2), GEN (4), SXT (>4), DOX (8), TGC (0.5)	23	56751	*bla*_CTX–M–65_, *bla*_TEM–1B_, *aac(3)-IV, aph(3″)-lb, aph(4)-Ia, aph(6)-Id, fosA3, mdf(A), sul1, sul2, tet(A), dfrA1*	ColpVC, FIB(A P001918), FIC(FII), I1
EM23-18-56	CTX (>32), FEP (>16), CIP (>2), GEN (>8), SXT (>4), DOX (8), CST (>4)	165	86233	*bla*_CTX–M–123_, ***mcr-1.1***, *bla*_OXA–1_, *bla*_TEM–1B_, *aac(3)-IV, aac(6′)-Ib-cr, aph(3″)-Ib, aph(4)-Ia, aph(6)-Id, fosA3, mdf(A), mph(A), catB3, floR, aac(6′)-Ib-cr, oqxA, oqxB, qnrS1, arr-3, sul1, sul2, tet(A), dfrA17*	FIB, FIC, HI2, HI2A, **I2(Delta)** (63 kb)
EM23-18-58	CTX (8), SXT (>4), DOX (8), TGC (0.5)	58	135977	*bla*_CTX–M–15_, *bla*_TEM–1B_, *aph(3″)-Ib, aph(6)-Id, mdf(A), qnrS1, sul2, tet(A), dfrA14*	FIB(A P001918), FIC(FII), HI2, HI2A, I2(Delta)
**Traveler, pre-trip**					
49-A	CST (>4)	327	89745	*mdf(A)*	FIB, FII
BS115-A	CST (>4)	73	9897	*mdf(A)*	nd
**Traveler, post-trip**					
BS115R-A	CST (>4)	73	9897	*mdf(A)*	nd
BS15R-A	CTX (16), FEP (4), CIP (2), DOX (>16)	278	1323	***bla*_CTX–M–14_**, *bla*_TEM–1B_, *fosA5, mdf(A), floR, qnrS1, sul2, tet(A)*	FIB, X1, **Y** (121 kb), Col440I
BS90R-A	CTX (>32), FEP (4), CIP (0.5), GEN (>8), DOX (16)	654	47983	***bla*_CTX–M–55_**, *aac(3)-IId, mdf(A), qnrS1, tet(A), tet(M)*	**HI1** (185 kb), FIB, Q1, X1, Col(pHAD28)
BS90R-D	SXT (>4), DOX (8), CST (>4)	34	63139	***mcr-1.1***, *bla*_TEM–1B_, *aadA1, aadA2, mdf(A), cmlA1, qnrS2, sul3, tet(M), dfrA12*	FIB, FII, N, Q1, X1. **X4** (33 kb), p0111
BS74R-A	CTX (16), CIP (0.5)	448	86655	***bla*_CTX–M–15_**, *mdf(A), qnrS1*	**K** (95 kb), p0111
BS74R-B	CTX (>32), FEP (8), CIP (0.5), GEN (>8), SXT (>4), DOX (>16), TGC (2)	11088	137881	*bla*_CTX–M–55_, *bla*_TEM–1B_, *aac(3)-IId, aadA24, mdf(A), mef(B), floR, qnrS1, sul2, sul3, tet(B), dfrA12*	Y
BS74R-D	SXT (>4), DOX (16), CST (>4)	10	95715	***mcr-1.1***, *bla*_TEM–1B_, *aadA2, mdf(A), floR, qnrS1, sul2, sul3, tet(A), tet(M), dfrA12*	FIA, FIB, FII, X1, **X4** (34 kb), Y, Col440I
16R-A	CTX (>32), FEP (16), SXT (>4)	38	140254	*bla*_CTX–M–15_, *aadA1, aph(3″)-Ib, aph(6)-Id, mdf(A), sul2, dfrA1*	FIB, FII
16R-B	CTX (>32), FEP (16), SXT (>4), CST (>4)	38	140254	*bla*_CTX–M–15_, *aadA1, aph(3″)-Ib, aph(6)-Id, mdf(A), sul2, dfrA1*	FIB, FII
16R-C	GEN (>8), SXT (>4), DOX (8), CST (>4)	11087	137880	***mcr-1.1***, *bla*_TEM–1B_, *aac(3)-IId, aadA5, aph(3″)-Ib, aph(6)-Id, mdf(A), mph(A), sul1, sul2, tet(A), dfrA17*	FIB, FII, **I2** (63 kb), Col156, ColpVC

*ST, sequence type; cgST, core-genome ST; nd, not detected; TZP, piperacillin/tazobactam; CTX, cefotaxime; FEP, cefepime; ETP, ertapenem; CIP, ciprofloxacin; GEN, gentamicin; SXT, trimethoprim/sulfamethoxazole; DOX, doxycycline; TGC, tigecycline; CST, colistin.*

*^*a*^Strains that also underwent WGS with Nanopore are underlined.*

*^*b*^MICs were interpreted according to the EUCAST (version v.9.0) for Enterobacterales and the CLSI (M100, Ed30E) for doxycycline. Only antimicrobials with non-susceptible MICs are shown (full results are shown in Supplementary Table 1).*

*^*c*^Replicon type(s) and main resistance genes that were found on the same plasmid sequence or contig are shown in bold.*

*^*d*^In strains EH8-18-36, BS90R-A and EH01-18-04-A *bla*_CTX–M–55_ was associated with an upstream IS*Ecp1*. In the remaining strains, *bla*_CTX–M_ was located on composite IS*26* transposons: EF7-18-58 (IS*26*-*bla*_CTX–M–55_-*wbuC*-ΔTn*3*-IS*26*), EM22-18-53 (IS*26*-*bla*_CTX–M–65_-*tnp*-ORF-IS*26*), EM06-18-14 (IS*26*-ORF-*tnp*-*bla*_CTX–M–65_-IS*5*-*tonB*-ORF-ORF-Tn*3*-ORF-IS*26*), and BS15R-A (IS*26*-ORF-*qnrS1*-IS*3*-ΔTn*3*-IS*Ecp1*-*bla*_CTX–M–14_-IS*5*-*tonB*-ORF-ORF-ORF-IS*26*). In strain EM18-18-42, *bla*_CTX–M–14_ was localized within an IS*5* composite transposon: IS*5*-IS*3*-Tn*3*-ORF-ORF-ΔTn*3*-*bla*_CTX–M–14_-IS*5*.*

*^*e*^Numbers in brackets refer to plasmid size.*

### Screening for *bla* and *mcr* Genes Along With Analysis of Clonality

From the overall collection of 109 AMR-*Ec*, a subset of 67 (61.5%) representative strains was selected from local people (*n* = 20; 14 ESC-R- and 6 CST-R-*Ec*), poultry (*n* = 18; 12 ESC-R- and 6 CST-R-*Ec*), and chicken meat (*n* = 17; 8 ESC- R-, 3 CST- R-, 5 ESC-/CST-R, 1 carbapenem-R-*Ec*), whereas all isolates detected from Swiss travelers (*n* = 12; 5 ESC- R-, 6 CST- R-, 1 ESC-/CST-R-*Ec*) were included. These 67 strains underwent microarray, *mcr-1* to *mcr-8* PCR, and rep-PCR analyses (Supplementary Table 1).

**FIGURE 1:**
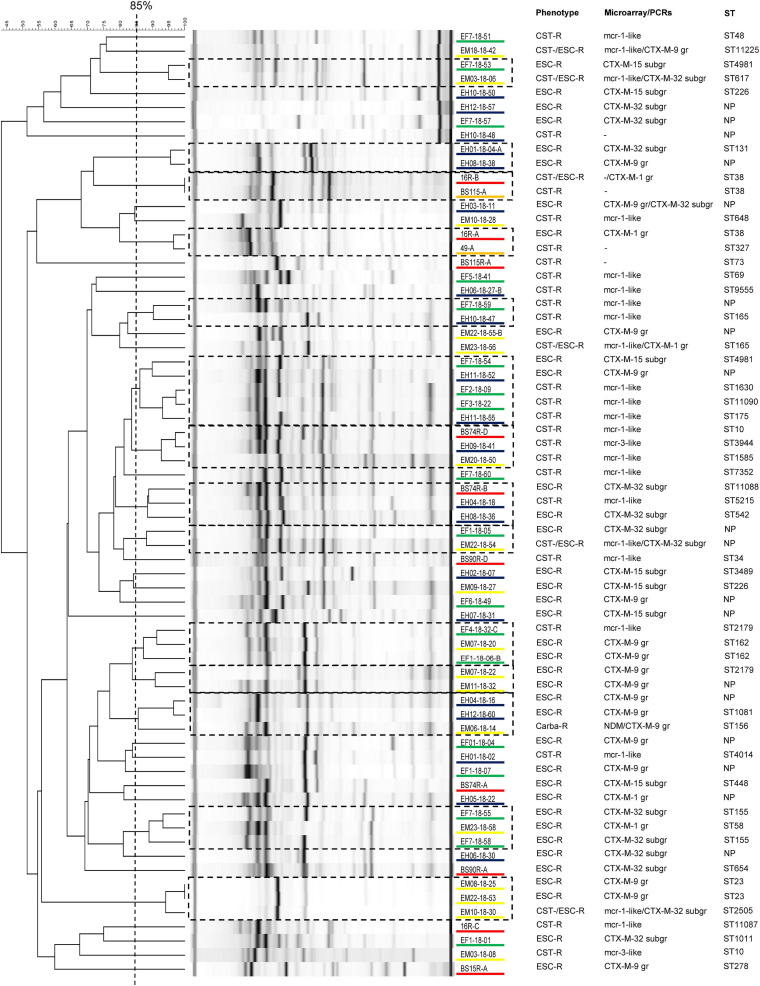
Analysis of the rep-PCR results from 67 *Escherichia coli* (*Ec*) strains isolated from local people (*n* = 20 out of 51 detected), poultry (*n* = 18 out of 28 detected), chicken meat (*n* = 17 out of 17 detected), Swiss residents after traveling to Laos (*n* = 10 out of 10 detected), and Swiss residents before traveling (*n* = 2 out of 3 detected). Strains showing an identity of ≥ 85% were considered to belong to the same clone. For each isolate, we show the phenotype, the main antimicrobial resistance genes (ARGs) detected by using microarray or PCRs, and the sequence type (ST). Isolates underlined in blue are from local people in Laos, in green from poultry, in yellow from chicken meat, in orange from Swiss residents before traveling, and in red from returning travelers. ESC-R, extended-spectrum cephalosporin-resistant; CST-R, colistin-resistant; Carba-R, carbapenem-resistant; NP, not performed (strains not typed with WGS); -, *mcr* and/or *bla*_ESBL_ genes not detected; gr, group; subgr, subgroup.

As shown in Figure 1, screening with microarray indicated that the 44 ESC-R-*Ec* (including the carbapenem-R strain) possessed only *bla*_CTX–M_ ESBL genes: *bla*_CTX–M–9group_ (*n* = 18; 40.9%), *bla*_CTX–M–32 subgroup_ (*n* = 15; 34.1%), *bla*_CTX–M–15 subgroup_ (*n* = 7; 15.9%), and *bla*_CTX–M–1group_ (*n* = 5; 11.4%). Furthermore, 24 (82.8%) out of the 29 CST-R-*Ec* harbored *mcr* genes (*mcr-1*-like, *n* = 22; *mcr-3*-like, *n* = 2).

Rep-PCR analysis also suggested a high heterogeneity among the isolates, with 30 (44.8%) of them being unique. The remaining 37 isolates clustered within 14 different clones with an average of only 2.6 strains each, indicating the absence of dominating clusters in our collection of AMR-*Ec* (Figure 1). This data was confirmed by the MLST, cgMLST and core-genome analyses (see below). We note that only one survey performed among pre-school children in Laos reported a certain clonality for the ESBL-producing *Ec* strains colonizing the gut (Stoesser et al., 2015).

### Characterization of ARGs

Forty-nine of the 67 AMR-*Ec* (45% of the total 109 detected strains) were also subjected to WGS by using Illumina. These AMR-*Ec* were from local residents (*n* = 11; 6 ESC-R- and 5 CST-R-*Ec*), poultry (*n* = 12; 6 ESC-R- and 6 CST-R-*Ec*), chicken meat (*n* = 14; 6 ESC- R-, 3 CST- R-, 4 ESC-R/-CST- R-, and 1 carbapenem-R-*Ec*), and travelers (all 12 strains; 5 ESC- R-, 6 CST- R-, 1 ESC-/CST-R-*Ec*).

As depicted in Table 2, ESC-R-*Ec* strains mostly possessed *bla*_CTX–M–15_ (*n* = 9; 31.0%) and *bla*_CTX–M–55_ (*n* = 9; 31.0%), which was consistent with a previous report from Laos (Stoesser et al., 2015). The majority of CST-R-*Ec* sourced in Laos and those from returning travelers carried *mcr-1.1*. Remarkably, *mcr-1.1* was found in *E. coli* strains isolated from all settings, suggesting a possible wide dissemination of common MGEs carrying *mcr-1.1* across many potential sources. This hypothesis is also supported by the many reports of *mcr-1*-harboring *Ec* strains from the same geographic area in human and non-human settings (Nang et al., 2019).

Notably, two *E. coli* strains sourced in Laos from a local person (EH09-18-41) and from a chicken meat sample (EM03-18-08) harbored the *mcr-3.1 and mcr-3.5* variants, respectively. We note that *mcr-3* (i.e., *mcr-3.21, mcr-3.26*, and *mcr-3.28* variants) has been reported from Laos only in *K. pneumoniae* strains isolated from the stools of healthy people collected in 2012 (Hadjadj et al., 2019).

### Comparison of AMR-*Ec* Obtained From Different Settings

To study the possible transmission or exchange of AMR-*Ec* among different settings, the above 49 representative strains also underwent MLST, cgMLST, and core-genome SNV analyses based on the Illumina outputs (Supplementary Table 3).

**FIGURE 2:**
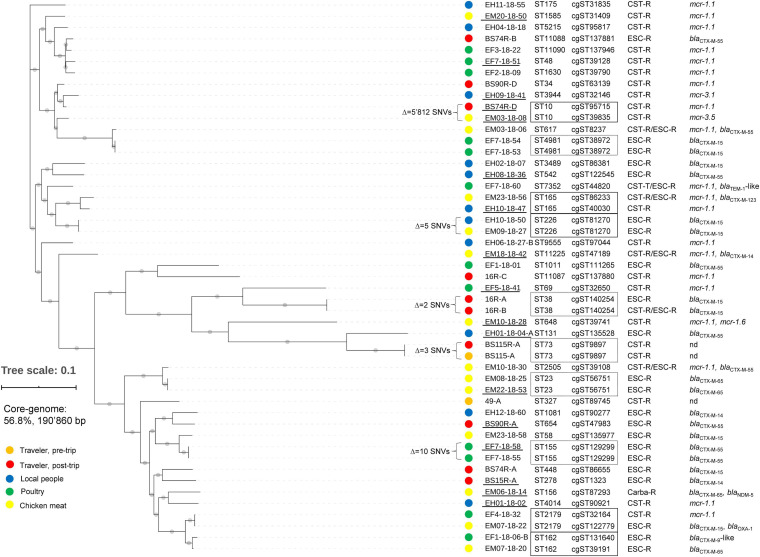
Results of the core-genome analysis for the 49 *E. coli* (*Ec*) strains isolated from travelers and local sources in Laos. The core-genome analysis of the assembled WGS of the strains is presented in a SNVs hierarchical clustering dendrogram tree. The Δ SNV values correspond to the number of non-identical SNVs between two strains. The SNV tree was visualized with iTOL (https://itol.embl.de). The filled colored circles on the left of the strain names indicate the different isolation sources: orange, red, blue, green, and yellow for traveler pre-trip, traveler post-trip, local people, poultry, and chicken meat, respectively. For each strain we also show the sequence type (ST), core-genome ST (cgST), and main resistance gene(s). CST-R: colistin-resistant, ESC-R: extended-spectrum cephalosporin-resistant, Carba-R: carbapenem-resistant, nd: not detected. Strain names that are underlined represent isolates for which the complete chromosome and plasmid sequences were obtained by a hybrid sequencing approach. Strains sharing the same ST are indicated with a dotted rectangle; those that were from different settings were indicated with a full rectangle.

As shown in Figure 2, among the overall 49 AMR-*Ec*, 39 different STs were found across all settings, of which 2 were from pre-trip traveler samples. Seven of the STs (ST131, ST648, ST38, ST10, ST48, ST69, and ST4014) were already reported in Laos (Stoesser et al., 2012, 2015; Olaitan et al., 2015), whereas the remaining 30 (of which 4 novel) were not previously described in the country. We emphasize that ST648 and ST131 are considered high-risk clones, but also others (e.g., ST10 and ST38) have been described worldwide in different settings (Peirano and Pitout, 2019).

Remarkably, 10 small groups of AMR-*Ec* (mostly pairs) shared the same ST, of which 5 included strains from different settings (indicated with rectangles in Figure 2). Nevertheless, our analysis showed that only two strains of ST226 were actually identical (both of cgST81270 and having Δ SNV = 5). Those two strains had been selected from a Laotian resident (EH10-18-50) and from a chicken meat sample (EM09-18-27) suggesting a possible transmission event of a CTX-M-15 producer from food to human or vice versa.

With regard to the nine travelers, we note that subject BS115 was colonized with the same CST-R ST73/cgST9897 strain (Δ SNV = 3) both pre- and post-trip. Thus, 4 (44.4%) travelers actually acquired AMR-*Ec* in Laos (i.e., BS15, 16, BS74, and BS90). In particular, these subjects were colonized with *bla*_CTX–M_-positive strains. Traveler BS74 also co-carried an *mcr-1.1*-positive *Ec* (BS74R-D) that shared the same ST10 with an isolate from chicken meat (EM03-18-08), but the two AMR-*Ec* had diverse cgSTs, *mcr* genes, and showed a Δ SNVs of 4’603 (Figure 2).

Overall, core-genome analysis, coupled by ST, cgST, and ARGs definition, suggested that no common AMR-*Ec* were spreading among the different local Laotian settings. More importantly, the analyzed local sources were not the origin for the transmission of specific AMR-*Ec* lineages to travelers. This data is in contrast to what we recently observed for Zanzibar (Tanzania), where identical AMR-*Ec* (e.g., the CTX-15-producing ST361) were exchanged among the local sources and were also acquired by the travelers (Budel et al., 2019, 2020; Moser et al., 2020). However, since spread and transmission of antimicrobial resistance could also occur via common MGEs, in the present work we performed a further analysis of plasmids and transposons carried by our AMR-*Ec* (see below).

### Genetic Background of *bla*_CTX–M_ Genes

In order to identify potential horizontal transfer of *bla*_CTX–M_- and/or *mcr*-harboring MGEs, among our 49 AMR-*Ec* that underwent Illumina WGS, we selected 17 archetypal isolates from local people (*n* = 4), poultry stool (*n* = 4), chicken meat (*n* = 6) and travelers (*n* = 3) for Nanopore long-read sequencing. Overall, 6 *bla*_CTX–Ms_-, 1 *bla*_CTX–M–14_–/*mcr-1*–, 1 *bla*_NDM–5–_/*bla*_CTX–M–65_–, 7 *mcr-1*–, and 2 *mcr-3*-possessing strains were analyzed (Table 2).

Of the 8 *bla*_CTX–M_-carrying isolates sequenced with both Nanopore and Illumina, 3 had a chromosomally-located *bla*_CTX–M_ and for 5 the gene was plasmidic. In particular, the *bla*_CTX–Ms_ were associated to three different plasmid types: IncHI1 (*n* = 3), IncFII and IncY (both, *n* = 1) (Table 2).

Briefly, the two IncHI1 *bla*_CTX–M–55_-carrying plasmids from traveler strain BS90R-A and from the resident strain EH08-18-36 showed an identity of ∼98.7–98.9% with a 253 kb *bla*_CTX–M–55_-positive plasmid isolated from an *Ec* of pig origin in Cambodia (GenBank: CP044299). The 210 kb *bla*_CTX–M–14_-carrying IncHI1 plasmid from chicken meat strain EM18-18-42 co-harbored *mcr-1.1* and was very similar to a plasmid found in Switzerland (discussed below in “Genetic Background of *mcr-1.1*” section) (Zurfluh et al., 2016). The 121 kb *bla*_CTX–M–14_-carrying IncY plasmid from traveler strain BS15R-A shared 98.9% identity with a 129 kb plasmid from a Chinese *Ec* (GenBank: MG196293), while the 82 kb *bla*_CTX–M–55_-carrying IncFII plasmid in poultry strain EF7-18-58 showed 99.9% identity with a 121 kb plasmid carried by an *Ec* found in France (GenBank: LT985277). Analysis of the Illumina WGS assemblies also revealed a further plasmid-located *bla*_CTX–M–15_ for the traveler strain BS74R-A (Table 2). Interestingly, this 95 kb IncK plasmid was almost identical (99% coverage and 98% identity) to a plasmid from an enterotoxigenic *Ec* that caused an outbreak in Korea in 2016 (data not shown) (Kim et al., 2017).

Notably, all *bla*_CTX–Ms_ were associated to known transposable elements (Poirel et al., 2012; Peirano and Pitout, 2019). In particular, two plasmid- and one chromosomally-located *bla*_CTX–M–55_ (hosted by human strains EH8-18-36, BS90R-A and EH01-18-04-A, respectively) were typically associated with an upstream IS*Ecp1* transposase. In the remaining five *bla*_CTX–M_-carrying strains, the gene was located on composite IS*26* transposons (see details in the note to Table 2).

### Genetic Background of *bla*_NDM–5_

The *bla*_NDM–5_ found in the chicken meat strain EM06-18-14 (ST156) was located in a 91 kb IncFII plasmid (pEM06-18-14_2; GenBank: CP063481) within a predicted IS*26* composite transposon: IS*26*-ΔIS*30*-*bla*_NDM–5_-*ble*-*iso*-ORF-IS*91*-*sul1*-*qacEΔ1*-ΔORF-IS*4*-*aadA2*-ORF-*dfrA12*-Δ*intI1*-IS*26*. A BLASTn search of the plasmid sequence showed that pEM06-18-14_2 displayed a high identity (99.9%) with pHNEC55, an 81 kb IncFII plasmid from an *Ec* isolated in China (GenBank: KT879914). The genetic context of the IS*26* composite transposon identified in pEM06-18-14_2 was also similar to the structure of the recently described IncFII plasmid pM505-NDM-5 from an *Ec* of human origin in Myanmar (GenBank: AP023236).

Notably, we recently identified the same structure in an IncFII plasmid from an ST167 *Ec* colonizing a healthy person in Switzerland, indicating the worldwide dissemination of this *bla*_NDM–5_-harboring transposon among different *Ec* strains (Endimiani et al., 2020). We also underline that NDM-5-producing *Ec* were previously reported in Laos only in human clinical samples (Cusack et al., 2019). Therefore, our finding might suggest human contamination and reinforces the urgent need to monitor antimicrobial resistance beyond the human settings.

### Genetic Background of *mcr-1.1*

Analysis of the Nanopore/Illumina hybrid assemblies revealed that *mcr-1.1* was plasmid-located in 6 AMR-*Ec* and chromosomally-harbored in two strains. Seven additional plasmid-located *mcr-1.1* were detected by analysis of the Illumina WGS assemblies. In total, *mcr-1.1* was identified on four different plasmid types: IncX4 (*n* = 5), IncI2 (*n* = 3), IncP1 (*n* = 3), and IncHI1 (*n* = 2) (Table 2). Notably, in previous studies, these *mcr-1.1*-carrying plasmids (*mcr-1.1-Cps*) have been shown to be conjugative (Zhao et al., 2017; Sadek et al., 2021).

**FIGURE 3:**
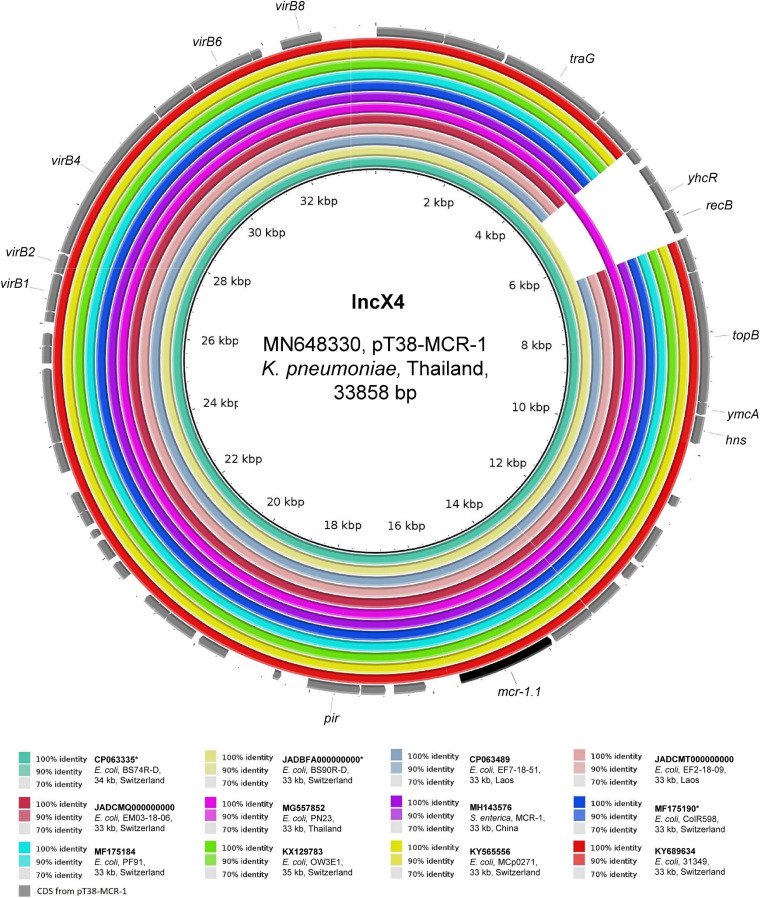
BLASTn comparison of IncX4-type plasmid sequences. The IncX4-type plasmid sequences from the present study were compared to other sequences that were selected based on high homology in a BLASTn search against the NCBI non-redundant nucleotide collection (on 10.11.2020). pT38-MCR-1 (GenBank accession: MN648330) was used as reference sequence. Rings were constructed using BRIG (BLAST Ring Image Generator) v.0.95. The colored rings represent similarities to the reference sequence. We report GenBank accession, species of isolation, strain name, sequence size and country of origin. CDS are represented as arrows in gray. The *mcr-1.1* gene is shown in black. The asterisk (*) indicates strains from Swiss residents with travel history to Asia.

As shown in Figure 3, the two ∼33 kb IncX4 *mcr-1.1*-Cp_*s*_ detected in travelers’ strains (BS74R-D and BS90R-D) were identical (100% coverage, > 99.9% identity) to two plasmids found in Thailand in *Ec* and *K. pneumoniae* strains from the stools of a duck and a healthy person, respectively (GenBank: MG557852 and MN648330) (Yu et al., 2020). Moreover, except for a 1,682-bp region that was missing in almost all of the other plasmid sequences, plasmids from our travelers were also identical to those from local sources in Laos (> 99.9% identity) and those found in other countries (e.g., including Switzerland and China). This observation is consistent with previous studies that reported little genetic variability between IncX4 *mcr-1.1*-Cp_*s*_ (Dona et al., 2017; Zurfluh et al., 2017; Nang et al., 2019).

**FIGURE 4:**
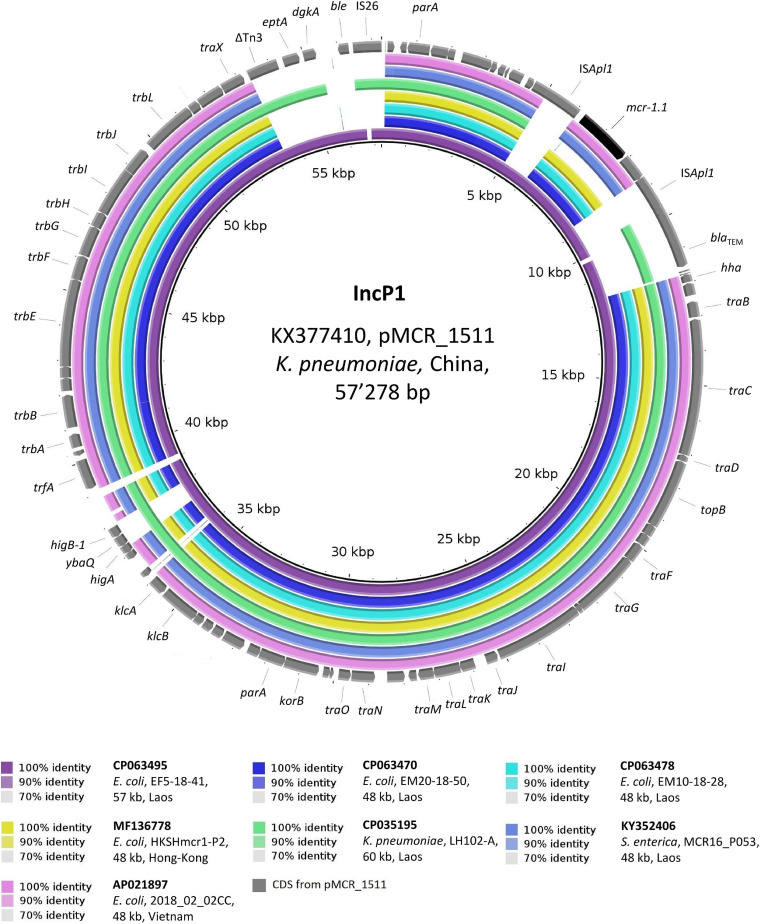
BLASTn comparison of IncP1-type plasmid sequences. The IncP1-type plasmid sequences from the present study were compared to other sequences that were selected based on high homology in a BLASTn search against the NCBI non-redundant nucleotide collection (on 10.11.2020). pMCR_1511 (GenBank accession: KX377410) was used as reference sequence. Rings were constructed using BRIG (BLAST Ring Image Generator) v.0.95. The colored rings represent similarities to the reference sequence. We report GenBank accession, species of isolation, strain name, sequence size and country of origin. CDS are represented as arrows in gray. The *mcr-1.1* gene is shown in black. Δ refers to truncated CDS.

As depicted in Figure 4, the 48 kb IncP1 *mcr-1.1*-Cp_*s*_ from chicken meat strains EM20-18-50 and EM10-18-28 were identical to each other and to plasmids from *Ec* isolated in both Hong-Kong and Vietnam from the stools of healthy people (overall, 99% coverage, >99.9% identity) (Chan et al., 2018). All of these IncP1 *mcr-1.1*-Cp_*s*_ also showed a high identity (98.6%) to the 57 kb plasmid (pEF5-18-41_3) originating from poultry strain EF5-18-41. Remarkably, pEF5-18-41_3 was the only plasmid in our study that harbored *mcr-1.1* which was flanked by two IS*Apl1* elements, making it identical (99.9% identity) to the plasmid sequence of pMCR_1511 (57 kb) from a Chinese *K. pneumoniae* isolated from hospital sewage (GenBank: KX377410) (Zhao et al., 2017).

Our 63 kb IncI2 *mcr-1.1*-Cp obtained from traveler’s strain 16R-C was similar (identity of ∼91-92%) to the two found in chicken meat strains EM10-18-30 and EM23-18-56 (Supplementary Figure 1), but almost identical (100% coverage, 99.9% identity) to a 67 kb plasmid recently described in a Vietnamese *Ec* isolated from food (GenBank: AP018355). In contrast to the Vietnamese plasmid, the MGEs from chicken meat and traveler strains were missing the IS*Apl1* upstream of *mcr-1.1* (Wang R. et al., 2018). Notably, similar 57–67 kb IncI2 *mcr-1.1*-Cp_s_ have been reported worldwide in various settings (Nang et al., 2019), including those in Switzerland (Dona et al., 2017; Zurfluh et al., 2017).

The 210 kb IncHI1 plasmid from chicken meat strain EM18-18-42 co-harbored *mcr-1.1* and *bla*_CTX–M–14_ and showed a high identity (99.9%) to a recently described plasmid (pH266B; 209 kb) found in Switzerland in a ST167 *Ec* isolated from vegetables imported from Thailand (Supplementary Figure 2) (Zurfluh et al., 2016). This plasmid was also the most similar to the other 227 kb IncHI1 *mcr-1.1*-Cp from the local person strain EH11-18-55 (99.8% identity). In both of our strains, *mcr-1.1* was located in the plasmid backbone with no surrounding transposases. This genetic context was also described for plasmid pH266B (Zurfluh et al., 2016).

Our results indicate that the different Laotian settings, but also those of the surrounding countries, may share very similar or even identical *mcr-1.1*-Cp_*s*_, thought they are hosted by different AMR-*Ec*. Moreover, international travelers may acquire such MGEs during their stay. Nevertheless, these *mcr-1.1*-Cp_*s*_ are nowadays also described in many continents (Nang et al., 2019). For instance, we found the same 33 kb IncX4 *mcr-1.1*-Cp in local poultry, chicken meat, and Swiss travelers, but this MGE was already described in Switzerland in humans, food, and environment (Figure 3) (Zurfluh et al., 2016, 2017; Dona et al., 2017). Therefore, it is difficult to understand what is the current extent and impact of the importation of these hyperepidemic plasmids on low-prevalence nations. However, it can be speculated that several years ago travelers visiting South-Asia were one of the main sources responsible for the importation, and subsequent diffusion in different settings, of the *mcr-1.1*-Cp_s_ to the low endemic countries such as Switzerland.

### Genetic Background of *mcr-3*

Two AMR-*Ec* harbored plasmid-mediated *mcr*-3 variants: strain EH9-18-41 (ST3944) from a local person and EM03-18-08 (ST10) from chicken meat (Figure 2 and Table 2).

EH9-18-41 hosted an IncFIB *mcr-3.1*-carrying plasmid with a genetic environment identical (100% coverage, 99.9% identity) to the recently described ΔIS*26*-ΔTn*As2*-*mcr-3.1*-*dgkA*-IS*Kpn40*-*ble*-IS*15DI* structure in plasmid pHN8 (GenBank: MG780294) from an *Ec* of pig origin in China (Wang Z. et al., 2018). In contrast, the IncFII plasmid in EM03-18-08 did not have a truncated IS*26* upstream of *mcr-3.5* and the IS*15DI* was located downstream of *dgkA*, similar to what was described for plasmid pGDZJ003-1-MCR-3 from an *Ec* found in China (GenBank: MH043625). Interestingly, the same genetic context has also been found in plasmid pT3 hosted by a CTX-M-55-producing *Ec* detected in a cricket sold as food in a Thai store in Switzerland (GenBank: MK656937) (Zurfluh et al., 2019). Finally, we note that the previously reported *mcr-3* variants from *K. pneumoniae* strains in Laos were associated to IncP1, IncFII, and IncI1 type plasmids (Hadjadj et al., 2019).

## Conclusion

This study has first contributed to fill up the scarcity of data regarding AMR in Laos. Our results indicate that the prevalence of AMR-*Ec* in people in the community, poultry, and chicken meat is high, but still does not reach the concerning rates reported from the neighboring countries.

We have also shown that the dissemination of *bla*_CTX–M_ and *mcr-1/-3* genes in this region occurs mostly through common MGEs rather than by clonal spread of specific strains. More importantly, we revealed that international travelers may locally acquire specific plasmids and import them to their home countries. However, since these MGEs are nowadays also described in many continents (in different settings and bacterial hosts) (Poirel et al., 2012; Nang et al., 2019), the real extent and impact of this phenomenon for low-prevalence countries cannot be defined.

As anticipated, our results about the mechanisms of AMR spread in Laos are in contrast with what we observed for Tanzania during a very similar survey (Budel et al., 2019, 2020; Moser et al., 2020). This difference (i.e., vertical vs. horizontal spread) can be attributed to the complexity of the AMR phenomenon that can follow different mechanisms and dynamics according to the diverse geographic areas. For these reasons, and to better understand this overall global issue, the WGS methodological approaches used in this study will be essential when surveying other countries. Moreover, in future studies, other potential sources (e.g., water and wildlife) that could play a major role in the dissemination of AMR should be explored (Kim et al., 2017).

Nevertheless, this study presents several limitations. First, for the local samples, we analyzed only one AMR-*Ec* colony obtained from the selective agar plates, which could have reduced the chance to observe possible exchange/transmission events of the same clones between different settings. Second, the sample size, in particular the number of participating travelers, represents only a small fraction of the sampled settings. It is possible that a clonal spread of AMR-*Ec* between these sources remained undetected because of the limited sample size. Future studies should address this issue by sampling a larger portion of the reservoirs. Third, the possible sources for AMR-*Ec* sampled in this study might not be the main sources responsible for transmission to travelers. Other reservoirs such as water, frequently touched surfaces, or further food items might be the main sources for colonization of travelers. Such possible sources should be analyzed in future studies. Finally, the time scale of the sampling in the different compartments was not fully identical, especially for travelers. Future studies on the persistence of AMR-*Ec* strains in the analyzed sources should face these limitations providing a better understanding on this issue.

## Data Availability Statement

The datasets presented in this study can be found in online repositories. The names of the repository/repositories and accession number(s) can be found in the article/Supplementary Material.

## Ethics Statement

The studies involving human participants were reviewed and ethics approval was obtained from the Kantonale Ethikkommission Zurich, the Ethics Committee Nordwest- und Zentralschweiz, and the Lao National Ethics Committee for Health Research (BASEC #: 2017-01945, NECHR #:2018-033). A written informed consent was obtained from all participants. The participants provided their written informed consent to participate in this study.

## Author Contributions

EK, CH, and AE: conception and design. AM, EK, EC-M, TB, SR, and MV: acquisition of the data. AM, EK, EC-M, TB, and AE: analysis and interpretation of the data. AM and AE: drafting the work. All authors involved in critical revision for important intellectual content, final approval of the version to be published, and agreement to be accountable for all aspects of the work in ensuring that questions related to the accuracy or integrity of any part of the work are appropriately investigated and resolved.

## Conflict of Interest

The authors declare that the research was conducted in the absence of any commercial or financial relationships that could be construed as a potential conflict of interest.

## Publisher’s Note

All claims expressed in this article are solely those of the authors and do not necessarily represent those of their affiliated organizations, or those of the publisher, the editors and the reviewers. Any product that may be evaluated in this article, or claim that may be made by its manufacturer, is not guaranteed or endorsed by the publisher.

## Footnotes

1 https://www.eucast.org/

2 https://check-pointshealth.com/user-manuals/

3 www.genomicepidemiology.org/

4 https://itol.embl.de
