# Supplementary material for: Antimicrobial-Resistant Escherichia coli Strains and Their Plasmids in People, Poultry, and Chicken Meat in Laos
Source: Front Microbiol. 2021 Jul 26;12:708182. doi: 10.3389/fmicb.2021.708182 (PMC8350485; doi:10.3389/fmicb.2021.708182)
Supplement: Supplementary file 2 [file Table_2.DOCX]

**Supplementary Table 2.** Demographic and clinical characteristics of the 9 travellers enrolled in the present study. Travelers who were colonized with antibiotic-resistant E. coli already before the trip have been highlighted in grey (n=2)

| **Group / Traveller** | **Age / Sex** | **Trip on:** | **Vegetarian** | **Use of antacids** | **In the last 12 months** | | | **Present trip in Laos** | | | | |
| --- | --- | --- | --- | --- | --- | --- | --- | --- | --- | --- | --- | --- |
|  |  |  |  |  | **Previous trips:**  **country (days of stay)** | **Hospitalized** | **Antibiotics** | **Days** | **Diarrhoea and severity** | **Other symptoms** | **Antibiotics** | **Medication(s)** |
| BS15 | 70 / F | 23.12.18 | - | - | IRL (4), ESP (4), PRT (8) | - | nd | 37 | +, medium | Na, Vom | - | Loperamide |
| 16 | 29 / F | 09.02.19 | - | - | ZAF (13), UGA (14), PRT (5), GER (7) | - | - | 8 | - | - | - | - |
| 49 | 57 / M | 05.02.19 | - | - | FRA (16), CHN (3), PRT (12), ESP (4), KHM (2), THA (2), LAO (16) | - | - | 15 | nd | nd | nd | nd |
| BS71 | 40 / F | 15.08.18 | - | + | PER (65), PRT (5), TZA (9) | - | +, TMP-SMX | 14 | - | - | - | - |
| BS74 | 60 / F | 16.11.18 | - | - | FRA (4), AUT (4), DE (2) | - | - | 24 | - | - | - | - |
| BS90 | 58 / F | 16.11.18 | - | - | FRA (2), GER (2), AUT (4) | - | - | 24 | - | - | - | - |
| BS115 | 40 / F | 25.11.18 | - | - | SWE (20) | - | - | 14 **^a^** | - | - | - | - |
| BS119 | nd / F | nd | - | - | AUS (16), USA (11), GER (8), NLD (10), FRA (6), NOR (1), DNK (1), ESP (1), GER (6) | + | - | nd | - | - | - | - |
| ZH93 | 52 / M | 07.04.19 | - | - | USA (14) | - | - | 23 | - | - | - | - |

**Note**. M, male; F, female; -, negative answer; +, positive answer; AUS, Australia; AUT, Austria; CHN, China; DNK, Denmark; ESP, Spain; FRA, France; GER, Germany; IRL, Ireland; KHM, Cambodia; NLD, Netherlands; NOR, Norway; PER, Peru; PRT, Portugal; SWE, Sweden; THA, Thailand; TZA, Tanzania; USA, United States of America; ZAF, South Africa; TMP-SMX, Trimethoprim/Sulfamethoxazole ; Na, nausea; Vom, vomiting; nd, not defined

^a^ She visited Laos for 14 days and then Cambodia for 14 days
