# Supplementary material for: Antimicrobial-Resistant Escherichia coli Strains and Their Plasmids in People, Poultry, and Chicken Meat in Laos
Source: Front Microbiol. 2021 Jul 26;12:708182. doi: 10.3389/fmicb.2021.708182 (PMC8350485; doi:10.3389/fmicb.2021.708182)
Supplement: Supplementary file 4 [file Presentation_1.PPTX]

## Slide 1
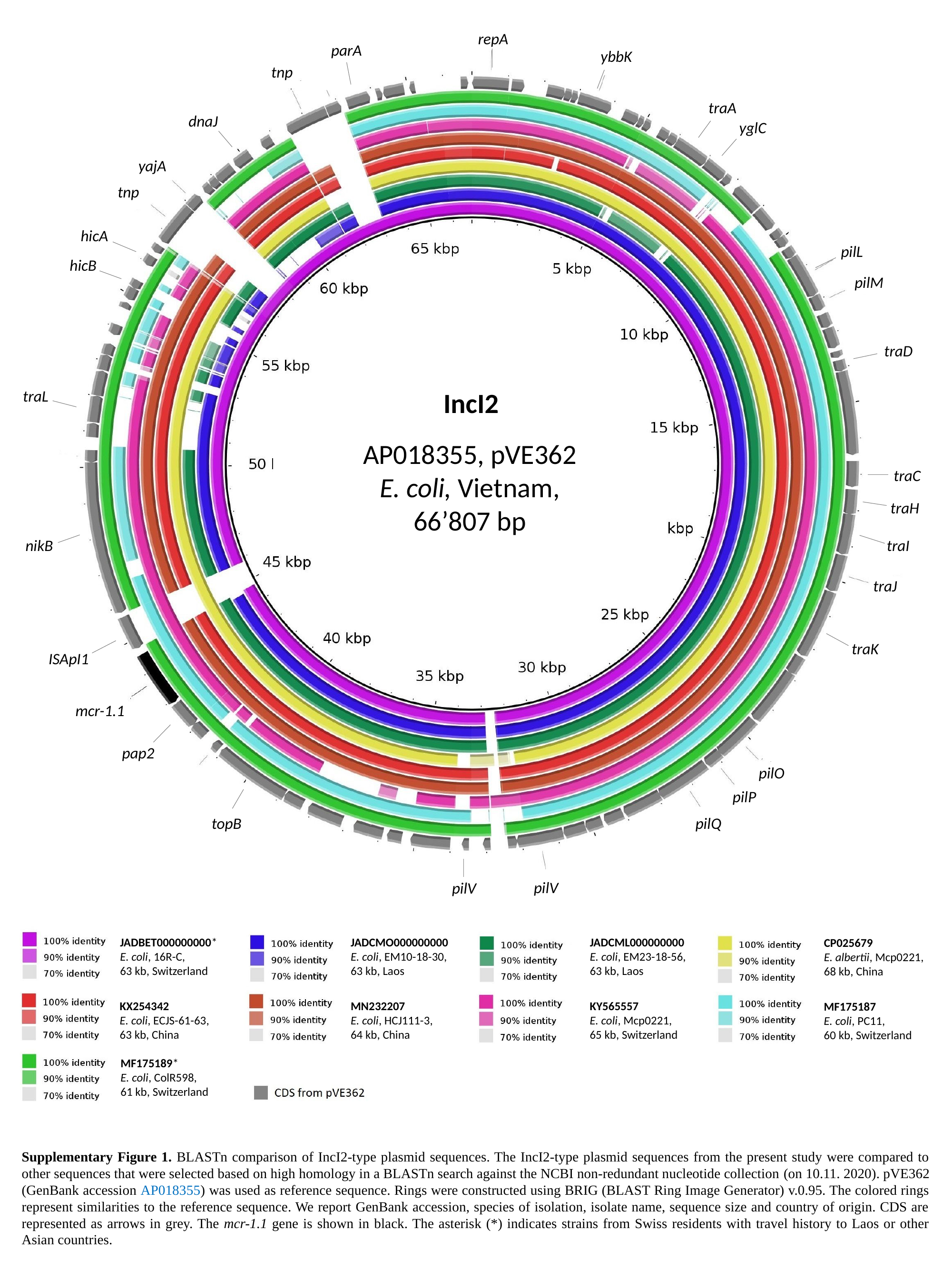

repA
parA
ybbK
tnp
traA
dnaJ
yglC
yajA
tnp
hicA
pilL
hicB
pilM
traD
IncI2
traL
AP018355, pVE362
E. coli, Vietnam,
66’807 bp
traC
traH
nikB
traI
traJ
traK
ISApI1
mcr-1.1
pap2
pilO
pilP
topB
pilQ
pilV
pilV
JADCMO000000000
E. coli, EM10-18-30,
63 kb, Laos
JADBET000000000*
E. coli, 16R-C,
63 kb, Switzerland
JADCML000000000
E. coli, EM23-18-56,
63 kb, Laos
CP025679
E. albertii, Mcp0221,
68 kb, China
MN232207
E. coli, HCJ111-3,
64 kb, China
KY565557
E. coli, Mcp0221,
65 kb, Switzerland
KX254342
E. coli, ECJS-61-63,
63 kb, China
MF175187
E. coli, PC11,
60 kb, Switzerland
MF175189*
E. coli, ColR598,
61 kb, Switzerland
Supplementary Figure 1. BLASTn comparison of IncI2-type plasmid sequences. The IncI2-type plasmid sequences from the present study were compared to other sequences that were selected based on high homology in a BLASTn search against the NCBI non-redundant nucleotide collection (on 10.11. 2020). pVE362 (GenBank accession AP018355) was used as reference sequence. Rings were constructed using BRIG (BLAST Ring Image Generator) v.0.95. The colored rings represent similarities to the reference sequence. We report GenBank accession, species of isolation, isolate name, sequence size and country of origin. CDS are represented as arrows in grey. The mcr-1.1 gene is shown in black. The asterisk (*) indicates strains from Swiss residents with travel history to Laos or other Asian countries.
