# Supplementary material for: Antimicrobial-Resistant Escherichia coli Strains and Their Plasmids in People, Poultry, and Chicken Meat in Laos
Source: Front Microbiol. 2021 Jul 26;12:708182. doi: 10.3389/fmicb.2021.708182 (PMC8350485; doi:10.3389/fmicb.2021.708182)
Supplement: Supplementary file 5 [file Presentation_2.PPTX]

## Slide 1
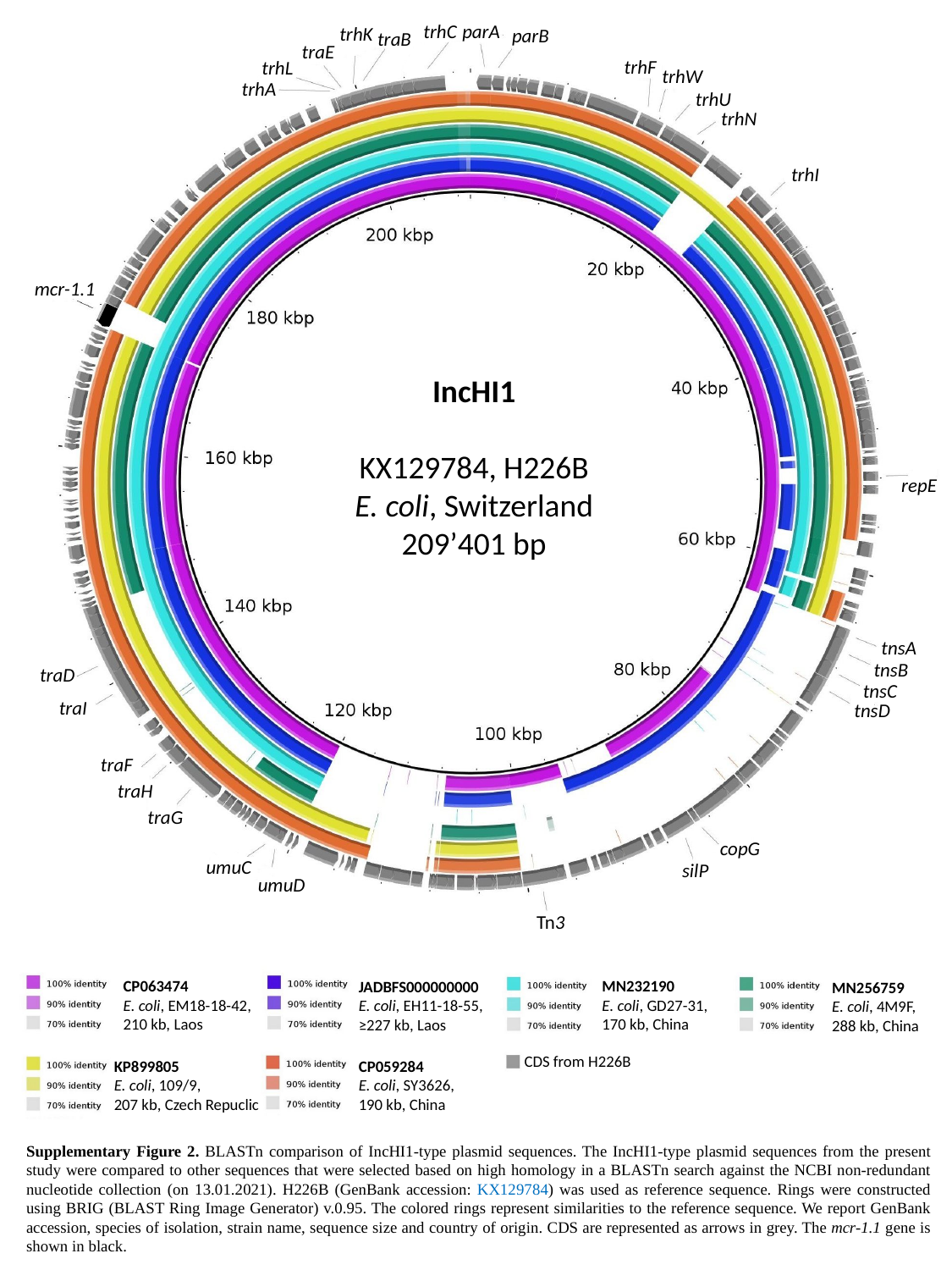

trhC
parA
trhK
parB
traB
traE
trhF
trhL
trhW
trhA
trhU
trhN
trhI
mcr-1.1
tnsA
tnsB
traD
tnsC
traI
tnsD
pap2
traF
traH
traG
copG
umuC
silP
umuD
Tn3
IncHI1
KX129784, H226B
E. coli, Switzerland
209’401 bp
repE
repE
MN232190
E. coli, GD27-31,
170 kb, China
CP063474
E. coli, EM18-18-42,
210 kb, Laos
JADBFS000000000
E. coli, EH11-18-55,
≥227 kb, Laos
MN256759
E. coli, 4M9F,
288 kb, China
CDS from H226B
CP059284
E. coli, SY3626,
190 kb, China
KP899805
E. coli, 109/9,
207 kb, Czech Repuclic
Supplementary Figure 2. BLASTn comparison of IncHI1-type plasmid sequences. The IncHI1-type plasmid sequences from the present study were compared to other sequences that were selected based on high homology in a BLASTn search against the NCBI non-redundant nucleotide collection (on 13.01.2021). H226B (GenBank accession: KX129784) was used as reference sequence. Rings were constructed using BRIG (BLAST Ring Image Generator) v.0.95. The colored rings represent similarities to the reference sequence. We report GenBank accession, species of isolation, strain name, sequence size and country of origin. CDS are represented as arrows in grey. The mcr-1.1 gene is shown in black.
